# Assessment of Benthic Ecological Quality Status in the Subtidal Zone of Northern Jeju Island, South Korea, During Summer Based on Macrobenthos

**DOI:** 10.3390/ani15040539

**Published:** 2025-02-13

**Authors:** Jian Liang, Chae-Woo Ma, Kwang-Bae Kim

**Affiliations:** 1 Department of Biology, College of Natural Sciences, Soonchunhyang University, Asan 31538, Republic of Korea; 2 Research Group of Tidal Flats, Gyeonggi-do Maritime and Fisheries Resources Research Institute, Ansan 15651, Republic of Korea

**Keywords:** southern sea of Korea, marine habitat conservation, benthic index, annelida, Jeju Strait

## Abstract

Background: Jeju Island is a world-renowned tourist destination. However, with the increasing intensity of anthropogenic activities, the coastal marine environment of Jeju Island has been negatively affected. Methods and Results: Our study used five benthic indices to assess the ecological quality of the subtidal zone in northern Jeju Island. Our analysis indicated that apart from the benthic polychaetes amphipods index (BPA), other benthic indices assessed the ecological quality of the subtidal zone in northern Jeju Island as high or good. Distance-based redundancy analysis (dbRDA) indicated that pH and salinity primarily influenced macrobenthic communities in 2011 and 2012. According to the Spearman rank correlation analysis, the multivariate AZTI marine biotic Index (M-AMBI) showed stronger correlations with environmental data than other benthic indices. Conclusions: Overall, the averages of the five benthic indices suggested that the benthic ecological quality of the subtidal zone in the northern part of Jeju Island was high or good. Although immediate marine environmental management is not required, long-term monitoring remains essential. M-AMBI outperformed other benthic indices; however, we recommend using multiple benthic indices to assess the benthic ecological quality of Jeju Island due to the complexity of marine ecosystems. Effective support has been provided by our study for the marine environmental conservation along the coast of Jeju Island.

## Simple Summary

The accurate assessment of benthic ecological quality is crucial for protecting marine environments. In our study, we used five benthic indices to evaluate the benthic ecological quality status of the northern coastal waters of Jeju Island. Our results indicated that the averages of the five benthic indices suggested that the benthic ecological quality of the subtidal zone in the northern part of Jeju Island was high or good. Although the multivariate AZTI marine biotic Index (M-AMBI) showed a stronger performance in correlation analyses compared to the other indices, we still recommend using multiple benthic indices to evaluate the benthic ecological quality status.

## 1. Introduction

The geographical isolation of islands has given rise to unique habitats that support a wide array of endemic species, significantly contributing to global biodiversity [1]. However, as the global population grows, island ecosystems have become increasingly threatened by anthropogenic activities [2,3]. While considerable research has been devoted to understanding the impact of these activities on terrestrial ecosystems within islands [4], there has been relatively little focus on their effects on island benthic ecosystems [5].

To assess the impact of anthropogenic activities on coastal ecosystems, researchers have developed various biological indices that are specifically designed to measure and monitor the health of these environments [6]. For instance, plankton and algae are commonly used to evaluate marine ecological quality [7,8]. Among these, macrobenthos-based benthic indices have gained widespread use in assessing coastal ecological quality [9]. Notably, the AZTI marine biotic Index (AMBI), which classifies macrobenthic organisms into different ecological groups according to their tolerance to organic matter, stands out as one of the most widely adopted and successful benthic indices globally [10].

Jeju Island, a world-renowned tourist destination, is approximately 90 km south of the Korean Peninsula [11]. Since the 1960s, with the rapid economic development of South Korea, various anthropogenic activities have significantly impacted the island’s ecological environment, notably tourism, agriculture, and aquaculture [12,13,14]. The expansion of these industries has profoundly affected Jeju’s natural resources and ecosystems, resulting in environmental degradation, reduced biodiversity, and increased water and soil pollution. The impact of anthropogenic activities on the coastal marine environment of Jeju Island has garnered widespread attention from researchers. Specifically, effluent from land-based aquaculture operations may contribute to organic pollution in coastal waters [15]. Furthermore, marine debris has reduced the density and biomass of macrobenthos along the coast of Jeju Island [16]. Compounding these issues, rising sea temperatures around Jeju Island have caused profound changes in benthic communities, including the seasonal absence of certain benthos [17]. Although two studies have utilized benthic indices, specifically the AMBI and Benthic Pollution Index (BPI), to assess the ecological quality of the waters surrounding Jeju Island, it is important to note that both studies relied exclusively on polychaetes for calculating these indices [18,19]. Relying on a single taxonomic group may not comprehensively assess ecological quality [20].

In our study, we selected the following five benthic indices: AMBI [21], Benthic index (BENTIX) [22], benthic polychaetes amphipods index (BPA) [23], BPI [24], and multivariate AZTI marine biotic Index (M-AMBI) [25]. These benthic indices have been widely used along the Korean coast to evaluate the ecological quality of the subtidal zone in South Korea. The five benthic indices have been extensively validated for their effectiveness in assessing ecological quality and responsiveness to anthropogenic pressures [26]. Our study provides a baseline for using benthic indices on Jeju Island and offers data support for the South Korean government in formulating coastal conservation policies for Jeju Island.

## 2. Materials and Methods

### 2.1. Study Area

**Figure 1:**
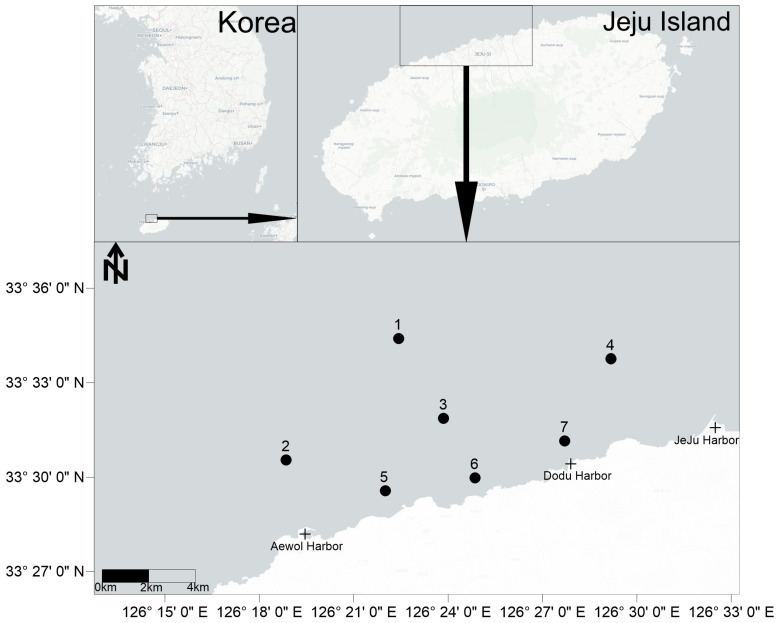
Study area and sampling stations (1–7) in the subtidal zone of northern Jeju Island.

The study area is located in the northern waters of Jeju Island. The study area extends from 33°34′24.1″ to 33°29′34.0″ N and from 126°18′50.8″ to 126°29′10.3″ E. Jeju Strait has a maximum depth exceeding 120 m. However, the study area was located along the coast of Jeju Island, where the water depth did not exceed 100 m. The total area of Jeju Island is about 1846 km^2^. There are three harbors within the study area: Aewol Harbor, Dodu Harbor, and Jeju Harbor (Figure 1). Awol Harbor and Dodu Harbor are fishing ports, while Jeju Harbor is a logistics and passenger port [27]. The annual average seawater temperature along the coast of Jeju Island is 18–20 °C [28]. In summer, precipitation and Changjiang Diluted Water can influence seawater salinity along Jeju Island’s coast [29,30]. The salinity of seawater along the coast of Jeju Island ranges from 23.94 to 34.97 psu [29].

### 2.2. Sample Collection and Processing

Sample collection was conducted in the northern waters of Jeju Island in August 2011 and September 2012, with seven sampling stations being strategically established (Figure 1). These stations were situated near ports and urbanized areas, representing the pressures imposed by human activities. During the summer, which is characterized by higher temperatures, benthic communities typically exhibit a greater diversity and abundance [31]. Surveys conducted in this season are particularly effective at capturing the characteristics of the community and assessing the benthic ecological quality of the subtidal zone of Jeju Island. At each station, a 0.1 m^2^ van Veen grab sampler was utilized to collect three samples—two for macrobenthos analysis and one for sediment analysis. The total sampling area for macrobenthic samples was 0.2 m^2^, while the sampling area for sediment samples was 0.1 m^2^.

To evaluate the characteristics of the benthic environment, the organic matter content in the sediment and the chemical properties of the bottom seawater were analyzed. In the field, macrobenthic samples were filtered using a 0.5 mm sieve with seawater and were then preserved in a 10% neutral formalin solution. Sediment samples were preserved at −40 °C and transported to the laboratory. At each station, bottom seawater samples were collected using a Niskin water sampler. A multiparameter water quality sonde (YSI 6920, YSI Inc., Yellow Springs, OH, USA) was used to analyze DO, pH, salinity, and water temperature.

In the laboratory, macrobenthic samples were identified to the species level using a versatile stereo microscope (SMZ-168, Motic Ltd., Xiamen, China). The structure of benthic communities is influenced by sediment type and its organic content [30]. To explore these relationships, a detection tube and titration methods were used to measure concentrations of acid volatile sulfide (AVS) and chemical oxygen demand (COD) in sediment samples. A wet pipetting method was used to calculate the mean grain size of sediment samples. A 10 g dry sediment sample was placed in a muffle furnace and heated to 550 °C for 2 h to calculate ignition loss (IL). Analysis methods for sediment samples strictly followed the Marine Environmental Process Test Standards [32].

### 2.3. Ecological and Benthic Indices

To evaluate the community characteristics of macrobenthos, four traditional ecological indices were employed: the species richness index (d), Pielou’s evenness index (J′), the Simpson index (1-Lambda′), and the Shannon–Wiener diversity index (H′) [33]. Formulae for calculating these diversity indices are shown in Table 1.

AMBI categorizes macrobenthos into five ecological groups based on their tolerance to organic matter [21]. BENTIX follows a similar principle to AMBI but divides macrobenthos into only three ecological groups [22]. BPA is based on the ratio of polychaetes to amphipods [23]. BPI classifies macrobenthos into four functional groups according to feeding type and life history [24]. M-AMBI is derived through a factor analysis of AMBI, the Shannon diversity index, and species richness [25]. The five benthic indices have been widely used to evaluate the coastal benthic ecological quality in South Korea.

Calculations for AMBI and M-AMBI were performed using the AMBI 6.0 version software (https://ambi.azti.es/, accessed on 15 January 2024). Reference condition settings for M-AMBI and functional group assignments for BPI were based on our previous research [34]. Formulae for calculating benthic indices are shown in Table 2.

### 2.4. Data Analysis

To assess the environmental characteristics of the subtidal zone in northern Jeju Island in 2011 and 2012, principal component analysis (PCA) was conducted on environmental data. Before analysis, data were transformed using log(x + 1) and normalized (Table 1). Spearman’s rank correlation analysis was performed to evaluate the relationships between benthic indices and environmental data. The environmental data, benthic communities, and the benthic ecological quality of the sampling stations in 2011 and 2012 were compared to comprehensively assess the annual differences. The Shapiro–Wilk test was initially employed to assess the normality, followed by either the paired *t*-test or the Mann–Whitney test to evaluate the differences among four diversity indices, five benthic indices, and environmental data. The distribution of macrobenthic communities around Jeju Island was visualized using non-metric multidimensional scaling (nMDS) analysis. The Analysis of Similarities (ANOSIM) was used to evaluate differences in benthic community composition. Distance-based redundancy analysis (dbRDA) was conducted using Bray–Curtis dissimilarity to investigate relationships between macrobenthic community dissimilarity and environmental data. PCA, nMDS, ANOSIM, and dbRDA were performed using PRIMER Version 7 software (PRIMER-E, NZL) [33]. Spearman’s rank correlation analysis, an Independent Samples *t*-test, and Mann–Whitney tests were conducted using OriginPro 2023 (OriginLab Inc., Northampton, MA, USA).

## 3. Results

### 3.1. Environmental Data on Jeju Island

**Figure 2:**
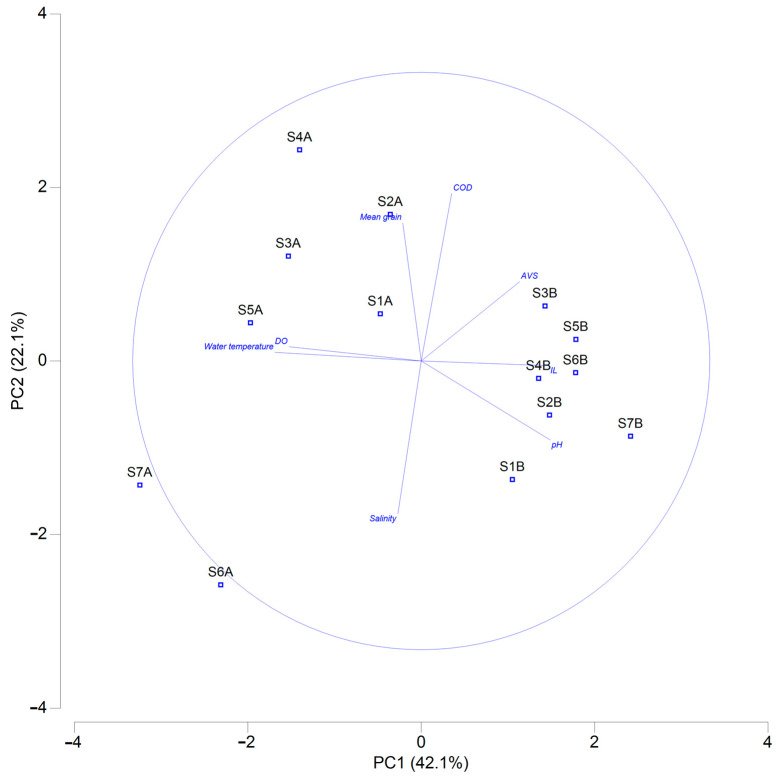
Principal component analysis of environmental data in the subtidal zone of northern Jeju Island. Note: AVS—acid-volatile sulfide; COD—chemical oxygen demand; DO—dissolved oxygen; IL—ignition loss; A—stations in 2011; B—stations in 2012.

The range and mean of environmental data are shown in Table 3. The coefficients of variation (CV) of AVS and the mean grain size were relatively higher than those of other environmental data. In the principal component analysis plot (Figure 2), the PC1 and PC2 axes explained 42.1% and 22.1% of the variance in environmental data, respectively. The PC1 axis exhibited the strongest correlations with water temperature (−0.507), dissolved oxygen (DO, −0.458), pH (0.447), and IL (0.442). The PC2 axis exhibited the strongest correlations with COD (0.581), mean grain size (0.478), and salinity (−0.530) (Table S1). These correlations suggest that PC1 primarily captured environmental gradients related to water chemistry and organic matter in sediments, while PC2 represented variations associated with salinity and sediment characteristics. Sampling stations in 2012 were primarily distributed on the right side of the principal component analysis plot. IL and pH were higher in 2012 than in 2011. The results of the paired *t*-test reveal significant annual variations in water temperature (t = 4.621, n1 = 7, n2 = 7, *p* = 0.004 two-tailed), DO (t = 3.528, n1 = 7, n2 = 7, *p* = 0.012 two-tailed), IL (t = −3.016, n1 = 7, n2 = 7, *p* = 0.024 two-tailed), and AVS (t = −3.576, n1 = 7, n2 = 7, *p* = 0.012 two-tailed), while the Mann–Whitney test indicates a significant annual variation in pH (Mann–Whitney U = 49, n1 = 7, n2 = 7, *p* = 0.002 two-tailed).

### 3.2. Macrobenthic Community Characteristics on Jeju Island

**Figure 3:**
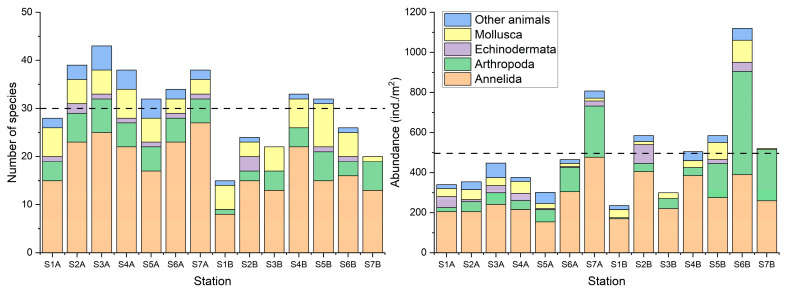
Number of species and abundance of species in the subtidal zone of northern Jeju Island. Note: A—stations in 2011; B—stations in 2012; broken line—average value.

**Figure 4:**
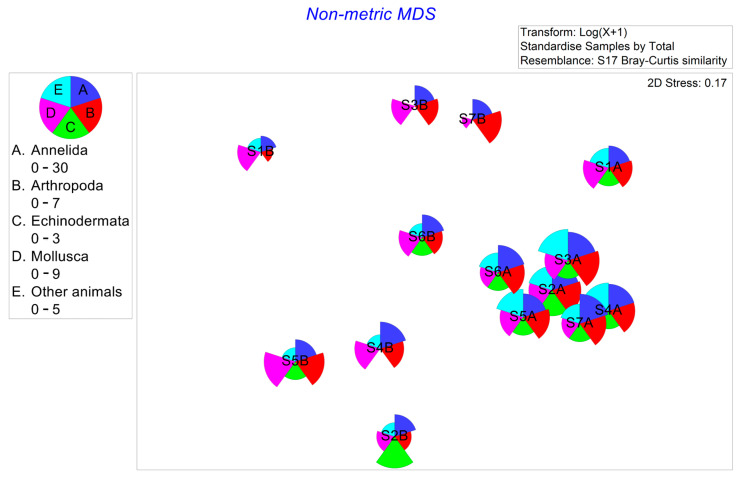
Non-metric multidimensional scaling ordination of macrobenthic communities in the subtidal zone of northern Jeju Island. Note: A—stations in 2011; B—stations in 2012.

In our study, a total of 101 macrobenthic taxa were identified, with Annelida being the most abundant (60 taxa), followed by Mollusca (18 taxa), Arthropoda (14 taxa), Echinodermata (3 taxa), and other animals (6 taxa) (Table S2). The average number of species in 2011 was 36 taxa, higher than that (27 taxa) in 2012. However, species abundance in 2011 was 380 ind./m2, lower than that (582 ind./m^2^) in 2012. The number of species at Station 1 in 2012 was the lowest (15 taxa), while Station 7 in 2011 had the highest number (44 taxa). Species abundance at Station 1 in 2012 was the lowest (235 ind./m^2^), whereas Station 6 in 2012 had the highest abundance (1120 ind./m^2^, Figure 3). The MDS plot revealed variations in macrobenthic communities relative to the sampling year, showing that most sampling stations from 2011 clustered closely together (Figure 4). The results of the ANOSIM also indicated significant annual differences in the macrobenthic communities of Jeju Island (R = 0.483; significance level of sample statistic = 0.1%).

### 3.3. Ecological and Benthic Indices on Jeju Island

**Figure 5:**
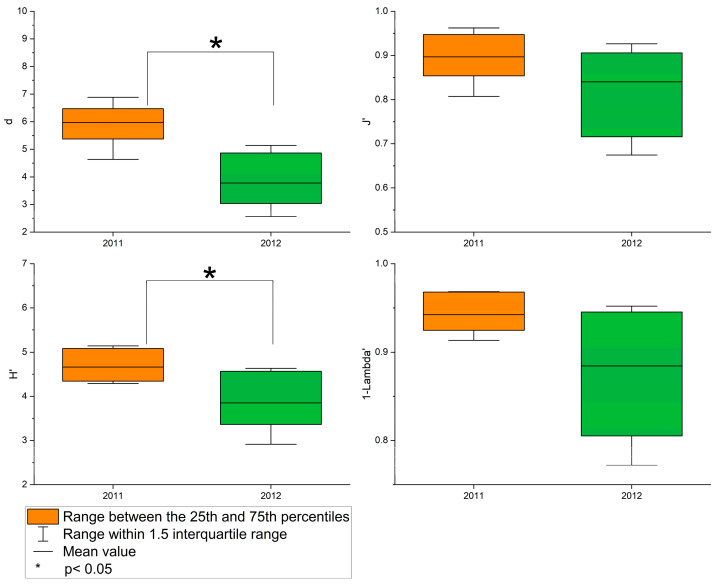
Box plot for ecological indices in the subtidal zone of northern Jeju Island.

The average values of the four ecological indices, d, J′, H′, and 1-Lambda′, were 5.97, 0.90, 4.66, and 0.94 in 2011 and 3.78, 0.84, 3.85, and 0.88 in 2012, respectively (Figure 4). The highest d and H′ values were 6.88 and 5.14, respectively, at Station 3 in 2011. The highest values of J′ and 1-Lambda′ were 6.88 and 5.14, respectively, at Station 3 in 2011. The lowest value of d was 2.56 at station 1 in 2012. The lowest values of J′, H′, and 1-Lambda′ were 0.67, 2.91, and 0.77, respectively, at Station 7 in 2012. The values of the four ecological indices at each station are shown in Figures S1–S4. The results of the paired *t*-test indicated that there was a significant annual difference in the value of d (t = 5.789, n1 = 7, n2 = 7, *p* = 0.001 two-tailed) and H′ (t = 4.011, n1 = 7, n2 = 7, *p* = 0.007 two-tailed) (Figure 5).

**Figure 6:**
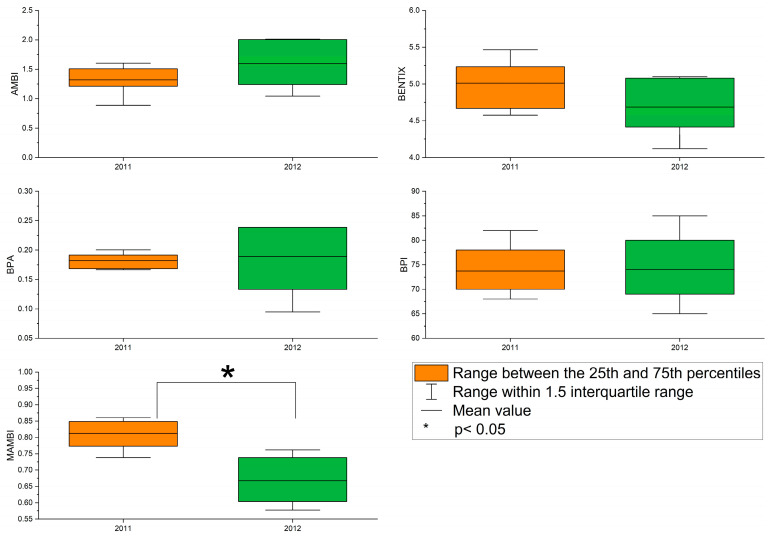
Box plot for benthic indices in the subtidal zone of northern Jeju Island.

The average values of the five benthic indices, AMBI, BENTIX, BPA, BPI, and MAMBI, were 1.32, 5.01, 0.18, 73.71, and 0.81 in 2011 and 1.59, 4.69, 0.19, 74.00, and 0.67 in 2012, respectively (Table 4). Except for BPA, other benthic indices (AMBI, BENTIX, BPI, and M-AMBI) evaluated the benthic ecological quality at all stations as either high or good in 2011 and 2012. The ecological quality of stations 1–4 in 2012 was assessed as moderate based on the BPA (Figure S7). The values of the five benthic indices and benthic ecological quality at each station are shown in Figures S5–S9. The results of the paired *t*-test indicated that there was a significant annual difference in the value of the M-AMBI (t = 5.171, n1 = 7, n2 = 7, *p* = 0.002 two-tailed) (Figure 6).

### 3.4. Results of Statistical Analysis

**Figure 7:**
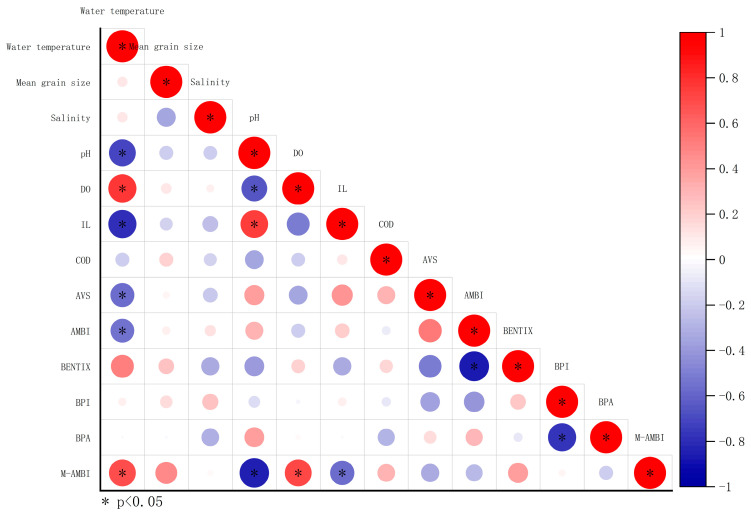
Heatmap of correlations between benthic indices and environmental data.

Spearman’s rank correlation analysis revealed negative correlations of AMBI with BENTIX and water temperature, a negative correlation of BPI with BPA, positive correlations of M-AMBI with water temperature and DO, and negative correlations of M-AMBI with pH and IL (Figure 7).

**Figure 8:**
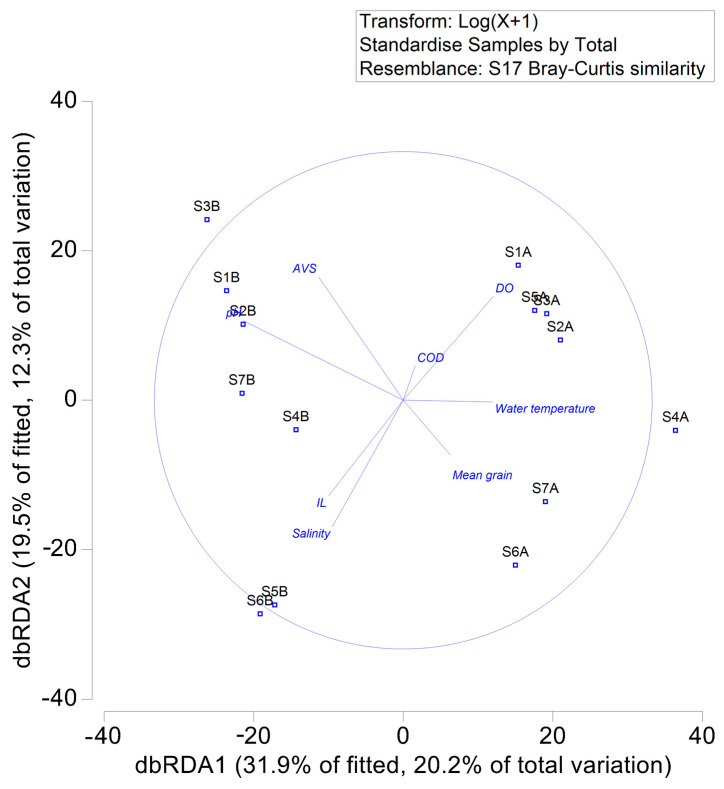
Distance-based redundancy analysis (dbRDA) diagram of the Bray–Curtis similarity index for the macrobenthic community. Note: A—stations in 2011; B—stations in 2012; AVS—acid-volatile sulfide; COD—chemical oxygen demand; DO—dissolved oxygen; IL—ignition loss.

The dbRDA analysis explained 51.4% of the variation in macrobenthic abundance, with the dbRDA1 and dbRDA2 axes accounting for 20.2% and 12.3% of the variation, respectively (Figure 8). The dbRDA1 axis was positively correlated with IL and mean grain size but negatively correlated with other environmental data. The dbRDA2 axis was negatively correlated with IL, mean grain size, salinity, and water temperature but positively correlated with other environmental data. The distribution of sampling stations showed that 2011 stations were all located on the right side of the figure, indicating that pH and salinity were the main environmental data contributing to distribution differences between 2011 and 2012 (Table S3).

## 4. Discussion

### 4.1. Environmental Characteristics on Jeju Island

In this study, concentrations of COD and IL in subtidal sediments of northern Jeju Island were lower than those in Asan Bay, Cheonsu Bay, and Gyeonggi Bay on the western coast of South Korea [36,37]. Compared to findings of Lee et al. (2022) [18], the average mean grain size in subtidal sediments of western and southern Jeju Island was significantly higher than that in the northern part of the island. In the correlation analysis, DO was positively correlated with water temperature. Our samples were collected during the summer, when higher water temperatures and stronger sunlight likely increased algal photosynthesis, leading to this positive correlation [38].

### 4.2. Macrobenthic Community on Jeju Island

The macrobenthic community in the subtidal zones of northern Jeju Island was predominantly composed of Annelida, whereas a previous study on the subtidal zone of southern Jeju Island (Munseom) found that the macrobenthic community was primarily dominated by Cnidaria [39]. This difference is likely attributed to the influence of sediment type, which plays a key role in shaping macrobenthic communities [30].

The average values of the four ecological indices were higher in August 2011 than in September 2012. The ANOSIM results further confirmed significant annual differences, demonstrating apparent variations in the macrobenthic community structure between 2011 and 2012. The dbRDA also indicated that pH and salinity were the main environmental data contributing to the differences in macrobenthic community structure between 2011 and 2012. The Kuroshio Current and Changjiang diluted water have the most significant impact on the coastal marine environment of Jeju Island in July and August [40]. Additionally, concentrated rainfall from the monsoon during these months can further influence coastal waters [41]. The Kuroshio Current, Changjiang Diluted Water, and summer precipitation might have caused seawater pH and salinity changes, leading to differences in macrobenthic community structure between 2011 and 2012. Notably, the non-metric MDS plot (Figure 4) shows that macrobenthic communities in 2011 were more clustered, while those in 2012 were more dispersed. In contrast, the PCA plot (Figure 2) of environmental data exhibits the opposite trend, with 2011 being more dispersed and 2012 being more clustered.

As previously discussed, the 2011 sampling occurred in August, when the influence of the Changjiang diluted water was at its strongest. However, the degree of this influence varied depending on the sampling site locations. In contrast, the 2012 sampling was conducted in September, by which time the influence of the Changjiang diluted water had weakened. This temporal difference likely explains the patterns observed in the PCA plot (Figure 2). However, due to their limited mobility, macrobenthic organisms are capable of reflecting long-term ecological changes in the region [42]. As a result, the strong influence of the Yangtze diluted water in August became evident in the macrobenthic communities sampled in September, leading to the observed differences in community structures.

### 4.3. Benthic Ecological Quality in Subtidal Zones of Northern Jeju Island

In the study by Liang et al. (2024) [37], a composite index integrating five benthic indices (AMBI, BENTIX, Benthic opportunistic polychaete amphipoda index, BPI, and M-AMBI) provided a more accurate assessment of benthic ecological quality in Korean bays than a single index. However, our study’s four benthic indices (AMBI, BENTIX, BPI, and MAMBI) yielded similar assessments, with all sampling stations being evaluated as high or good. We believe that when only one benthic index produces a differing assessment from the other four, a composite index may not be necessary. For instance, in 2012, the BPA rated the benthic ecological quality at Stations 1–4 as moderate, while the other indices rated it as good or high. This suggests that the overall benthic ecological quality in the subtidal zone of northern Jeju Island is acceptable. A composite index is useful for resolving conflicting classifications from different benthic indices. However, a composite index might not be required when the results are clear and consistent. Based on the concept of the composite index, we propose a practical rule: when the evaluation results of four indicators are uniformly “Good” or “High”, and only one indicator has an evaluation result lower than “Good”, we can reasonably consider the overall benthic ecological environment quality to be “Good”.

Due to their simple calculations, indices based on the ratio of polychaetes and amphipods, such as BPA, Benthic Opportunistic Polychaetes Amphipods index (BOPA), and Benthic Opportunistic Annelida Amphipods index (BO2A), have been widely used to assess coastal benthic ecological quality [43]. However, some studies have indicated that BOPA reflects human pressure on offshore platforms more effectively than AMBI and M-AMBI [44]. However, it is important to note that indices such as BPA only consider two taxonomic groups—polychaetes and amphipods. The absence of either taxonomic group can lead to inaccurate assessments of benthic ecological quality [45]. Specifically, the reason why the benthic ecological quality at Stations 1–3 in 2012 was assessed as moderate by the BPA was due to the absence of the amphipod taxonomic group. Thus, to avoid potential inaccuracies, indices based on the ratio of polychaetes and amphipods should be used with other benthic indices.

In the Spearman rank correlation analysis, correlations between environmental data and the benthic indices were generally weak except for the M-AMBI. Ecological group assignments in AMBI are based on European coastal ecosystems. However, the behavior and lifestyle of the same species can vary in different geographic regions [46]. Some studies have indicated that reassigning ecological groups based on a specific ecosystem of the study area can improve the performance of AMBI [47].

BENTIX assigns calculation weights to ecological groups based on the benthic community structure of the Mediterranean [48]. Due to the oligotrophic environment of the Mediterranean, the distribution of macrobenthic organisms is relatively uniform, with few species having an abundance more than 10% of the total abundance [49]. In northern Jeju Island, *Amphicteis gunneri* and Gammaridea abundances accounted for 14% and 10% of the total abundance, respectively. The use of BENTIX in the coastal areas of Jeju Island should be approached with caution. Additionally, some studies have pointed out that BENTIX is not suitable for the East Sea of Korea [50].

BPI is designed based on historical survey data from bays of the western coast of Korea. It is modeled after the ITI index [51]. In the study of Ryu et al. (2016) [51], BPI overestimated benthic ecological quality compared to other benthic indices on Korean coasts. However, the study by Liang et al. (2024) indicated that BPI underestimated the ecological quality in the tidal flats of Garolim Bay, Korea [52]. A notable challenge in using BPI is the need for an ecological group database similar to AMBI, which necessitates the involvement of researchers with specialized knowledge for accurate calculations. Given these inconsistencies and the complexities involved, we recommend that BPI should be used with other benthic indices, mainly when applied in open sea areas, to ensure a more reliable assessment of benthic ecological quality.

Overall, M-AMBI correlates more strongly with environmental data than other benthic indices. The dbRDA analysis observed that pH significantly influenced the macrobenthic community structure, with a negative correlation found between pH and M-AMBI. This index considers the number of species and the diversity of macrobenthos in its calculation. It was particularly effective in capturing changes in the macrobenthic community structure, making it more sensitive than other benthic indices [53]. Moreover, some studies have suggested that M-AMBI generally outperforms univariate benthic indices [33]. For instance, compared to AMBI, M-AMBI was more suitable for assessing the benthic ecological quality of the Yangtze River estuary [54]. It is important to note that M-AMBI has limitations. First, as previously mentioned, ecological group assignments in M-AMBI are based on European coastal ecosystems. Secondly, the performance of M-AMBI is highly dependent on reference condition settings, which are crucial for its effectiveness [55]. Additionally, some studies have pointed out that M-AMBI has a low salinity bias and that it tends to overestimate benthic ecological quality in sandy beach environments [56,57].

In addition, the paired *t*-test, the Mann–Whitney test, and ANOSIM results indicated significant differences in both the environment and benthic communities between 2011 and 2012. It is clear that these environmental disparities led to alterations in the benthic communities. However, remarkably, the benthic ecological quality status remained high or good throughout these two years. As elaborated in the text, the substantial differences observed in the benthic communities between 2011 and 2012 can likely be attributed to the influence of freshwater input from the Yangtze River. These natural interannual shifts in community composition, while notable, did not have a negative impact on the overall ecological quality of the northern waters of Jeju Island. This suggests that despite changes in macrobenthic community structure, the benthic ecosystem maintained its health.

### 4.4. Recommendations for the Use of Benthic Indices on the South Korean Coast

Although M-AMBI demonstrated a better performance than the other indices in our study, using multiple indices to assess benthic ecological quality is necessary, especially in regions where benthic indices have not been previously applied. Due to the complexity of marine ecosystems, it is challenging for a single benthic index to assess benthic ecological quality accurately [58]. Most studies have highlighted that different benthic indices perform differently in various regions [59]. However, different benthic indices within the same area may result in conflicting classifications of benthic ecological quality [60]. When multiple benthic indices provide conflicting classifications of benthic ecological quality, we recommend the use of a composite index, which can effectively avoid contradictory classifications.

The South Korean government has established clear quality standards for seawater and sediment in its coastal environmental management efforts. However, standards for assessing marine ecological quality based on macrobenthic indicators have not been approved yet for various reasons. Our study can serve as a reference for the South Korean government in advancing the establishment of standards for using macrobenthic indicators on the South Korean coast.

## 5. Conclusions

In our study, five benthic indices (AMBI, BENTIX, BPA, BPI, and MAMBI) were used to assess the benthic ecological quality of the sublittoral zone in the northern part of Jeju Island, South Korea. dbRDA analysis indicated that pH and salinity were the primary environmental data driving differences in macrobenthic community structure between 2011 and 2012. Except for the BPA, other benthic indices assessed the benthic ecological quality as high or good. Overall, the averages of the five benthic indices suggested that the benthic ecological quality of the subtidal zone in the northern part of Jeju Island was high or good. Although immediate marine environmental management is not required, long-term monitoring remains essential. This study not only established a baseline for using benthic indices on the coast of Jeju Island, but also provided valuable data for formulating related policies and for the conservation of the marine environment for the South Korean government.

## Figures and Tables

**Table 1:** The formulae for calculating the indices (five ecological indices and Bray–Curtis dissimilarity), algorithm, and note.

Indices	Algorithm	Note
Species richness index (d)	=(S−1)/log⁡(N)	S: the total number of species; N: the number of individual organisms.
Pielou’s evenness index (J′)	$=H^{’}/log(S)$	H′: Shannon–Wiener diversity index; S: the total number of species.
Simpson index (1-Lambda′)	$=1-SUM\left({Ni}^{*}(Ni-1)/\left(N^{*}(N-1)\right)\right.$	Ni: number of individuals of the ith species; N: the number of individual organisms.
Shannon–Wiener diversity index (H′)	$=-\sum \left[\left(\frac{n_{i}}{N}\right){log}_{2}\left(\frac{n_{i}}{N}\right)\right]$	Ni: number of individuals belonging to the ith species; N: total number of individuals.
Normalized calculation	$=\frac{X-\mu }{\sigma }$	X: original value; μ: mean value; σ: standard deviation of the dataset.
Bray–Curtis dissimilarity	$=100\frac{\sum_{j=1}^{p}\;\left|y_{ig}-y_{sk}\right|}{\sum_{i=1}^{p}\;\left(y_{ij}+y_{ik}\right)}$	S: the total number of species in the sample; $y_{ig}-y_{sk}$: the absolute difference in the abundance of species i between sample j and sample k; $y_{ij}+y_{ik}$: the sum of the abundances of species i in samples j and k.

**Table 2:** List, algorithms, index values, ecological quality status (EcoQs) thresholds, references, and explanatory notes of benthic indices.

Indices	Algorithm	Index Values	EcoQs	Reference	Note
AMBI	$\begin{array}{c} =[(0\times \%EGI)+(1.5\times \%EGII)+(3\times \%EGIII)\\ +(4.5\times \%EGIV)(6\times \%EGV)]/100 \end{array}$	0.0–1.2	High	[21]	EGI = disturbance-sensitive species; EGII = disturbance-indifferent species; EGIII = disturbance-tolerant species; EGIV = second-order opportunistic species; EGV = first-order opportunistic species.
		1.2–3.3	Good		
		3.3–5.0	Moderate		
		5.0–6.0	Poor		
		>6.0	Bad		
BENTIX	=[6×% G1+2(%G2+% G3)]/100	6–4.5	High	[22]	GI = EGI + EGII; GII = EGIII + EGIV; GIII = EGV.
		4.5–3.5	Good		
		3.5–2.5	Moderate		
		2.5–2.0	Poor		
		0.0	Bad		
BPA	=log⁡[(fP)/(fA+1)+1)]	0–0.135	High	[23]	fP = the frequency of polychaetes; fA = the frequency of amphipods.
		0.135–0.211	Good		
		0.211–0.26	Moderate		
		0.26–0.3	Poor		
		>0.3	Bad		
BPI	$\begin{array}{c} =[1-(a\times N1+b\times N2+c\times N3+d\times N4)/(N1+\\ N2+N3+N4)/d]\times 100 \end{array}$	60–100	High	[24]	N1 = filter feeders or large carnivores; N2 = surface deposit feeders or small carnivores; N3 = subterranean deposit feeders; N4 = opportunistic species; a = 0; b = 1; c = 2; d = 3.
		40–60	Good		
		30–40	Moderate		
		20–30	Poor		
		0–20	Bad		
M-AMBI	$=K+(a\times AMBI)+\left(b\times H^{’}\right)+(c\times S)$	>0.77	High	[35]	H′ = Shannon diversity index; S = number of species; a, b, and c = the coefficients derived from the factor analysis.
		0.53–0.77	Good		
		0.38–0.53	Moderate		
		0.20–0.38	Poor		
		≤0.2	Bad		

**Table 3:** Range, mean, and coefficient of variation of environmental data on Jeju Island.

Environmental Data	Range (Min–Max)	Mean ± CV
AVS, mg/g	0–0.004	0.002 ± 0.592
COD, g/Kg	3.07–7.19	4.95 ± 0.25
DO, mg/L	6.87–7.34	7.13 ± 0.02
IL, %	3.34–5.10	4.24 ± 0.15
Mean grain size, ø	0.90–4.20	2.16 ± 0.45
pH	7.86–8.23	8.11 ± 0.02
Salinity, PSU	32.41–33.56	33.12 ± 0.01
Water temperature, °C	20.3–23.5	21.91 ± 0.05

Note: AVS—acid-volatile sulfide; COD—chemical oxygen demand; DO—dissolved oxygen; IL—ignition loss.

**Table 4:** Range, mean, coefficient of variation, and EcoQs of benthic indices on Jeju Island.

Year	Benthic Index	Range (Min–Max)	Mean ± CV	EcoQs (Mean)
2011	AMBI	0.89–1.60	1.32 ± 0.18	Good
	BENTIX	4.58–5.47	5.01 ± 0.07	High
	BPA	0.17–0.20	0.18 ± 0.07	Good
	BPI	68.00–82.00	73.71 ± 0.07	High
	M-AMBI	0.74–0.86	0.81 ± 0.05	High
2012	AMBI	1.04–2.01	1.59 ± 0.23	Good
	BENTIX	4.12–5.10	4.69 ± 0.08	Good
	BPA	0.09–0.24	0.19 ± 0.32	Good
	BPI	65.00–85.00	74.00 ± 0.10	High
	M-AMBI	0.58–0.76	0.67 ± 0.10	Good

## Data Availability

The raw data supporting the conclusions of this article will be made available by the authors on request.

## Supplementary Materials

The following supporting information can be downloaded at: https://www.mdpi.com/article/10.3390/ani15040539/s1. Figure S1: Values of d at each station.; Figure S2: Values of J′ at each station; Figure S3: Values of H′ at each station; Figure S4: Values of 1-Lambda′ at each station; Figure S5: Values of AMBI and benthic ecological quality at each station; Figure S6: Values of BENTIX and benthic ecological quality at each station; Figure S7: Values of BPA and benthic ecological quality at each station; Figure S8: Values of BPI and benthic ecological quality at each station; Figure S9: Values of MAMBI and benthic ecological quality at each station; Table S1: Eigenvectors of environmental data with PC1 and PC2; Table S2: Abundance of species at each station; Table S3: Eigenvectors of environmental data to the dbRDA axes 1 and 2.
