# Supplementary material for: Assessment of Benthic Ecological Quality Status in the Subtidal Zone of Northern Jeju Island, South Korea, During Summer Based on Macrobenthos
Source: Animals (Basel). 2025 Feb 13;15(4):539. doi: 10.3390/ani15040539 (PMC11851414; doi:10.3390/ani15040539)
Supplement: Supplementary file 1 [file animals-15-00539-s001.zip › Supplementary Material (animals-3316427)-edit.pdf]

Supplementary Material

Assessment of Benthic Ecological Quality in the Subtidal Zone of Northern Jeju Island, South Korea, During Summer Based on Macrobenthic Indices

Jian Liang[1], Chae-Woo Ma[1, *] and Kwang-Bae Kim[2]

[1] Department of Biology, College of Natural Sciences, Soonchunhyang University, Asan 31537, Republic of Korea,

[2]Research Group of Tidal Flats, Gyeonggi-do Maritime and Fisheries Resources Research Institute, Ansan 15651, Republic of Korea

Content

Table S1. Eigenvectors of environmental data with PC1 and PC2. 2
Table S3. Eigenvectors of environmental data to the dbRDA axes 1 and 2. 3
Figure S1. Values of d at each station. Note: A—stations in 2011; B—stations in 2012; broken line—mean value. 4
Figure S2. Values of J′ at each station. Note: A—stations in 2011; B—stations in 2012; broken line—mean value. 5
Figure S3. Values of H′ at each station. Note: A—stations in 2011; B—stations in 2012; broken line—mean value. 6
Figure S4. Values of 1-Lambda' at each station. Note: A—stations in 2011; B—stations in 2012; broken line—mean value. 7
Figure S5. Values of AMBI and benthic ecological quality at each station. Note: A—stations in 2011; B—stations in 2012; broken line—mean value. 8
Figure S6. Values of BENTIX and benthic ecological quality at each station. Note: A—stations in 2011; B—stations in 2012; broken line—mean value. 9
Figure S7. Values of BPA and benthic ecological quality at each station. Note: A—stations in 2011; B—stations in 2012; broken line—mean value. 10
Figure S8. Values of BPI and benthic ecological quality at each station. Note: A—stations in 2011; B—stations in 2012; broken line—mean value. 11
Figure S9. Values of M-AMBI and benthic ecological quality at each station. Note: A—stations in 2011; B—stations in 2012; broken line—mean value. 12

Table S1. Eigenvectors of environmental data with PC1 and PC2.

Variable	PC1	PC2
AVS	0.341	0.275
COD	0.106	0.581
DO	-0.458	0.049
IL	0.442	-0.016
Mean grain size	-0.064	0.478
pH	0.447	-0.274
Salinity	-0.081	-0.530
Water temperature	-0.507	0.030

Note: AVS—acid-volatile sulfide; COD—chemical oxygen demand; DO—dissolved oxygen; IL—ignition loss.

Table S3. Eigenvectors of environmental data to the dbRDA axes 1 and 2.

Variable	dbRDA1	dbRDA2
AVS	-4.03	7.53
COD	-7.95	13.50
DO	-6.23	32.43
IL	1.10	-25.23
Mean	1.14	-6.01
pH	-24.43	34.30
Sal	-9.66	-9.13
Water temperature	2.64	-11.43

Note: AVS—acid-volatile sulfide; COD—chemical oxygen demand; DO—dissolved oxygen; IL—ignition loss; Sal—Salinity; Mean—mean grain size.

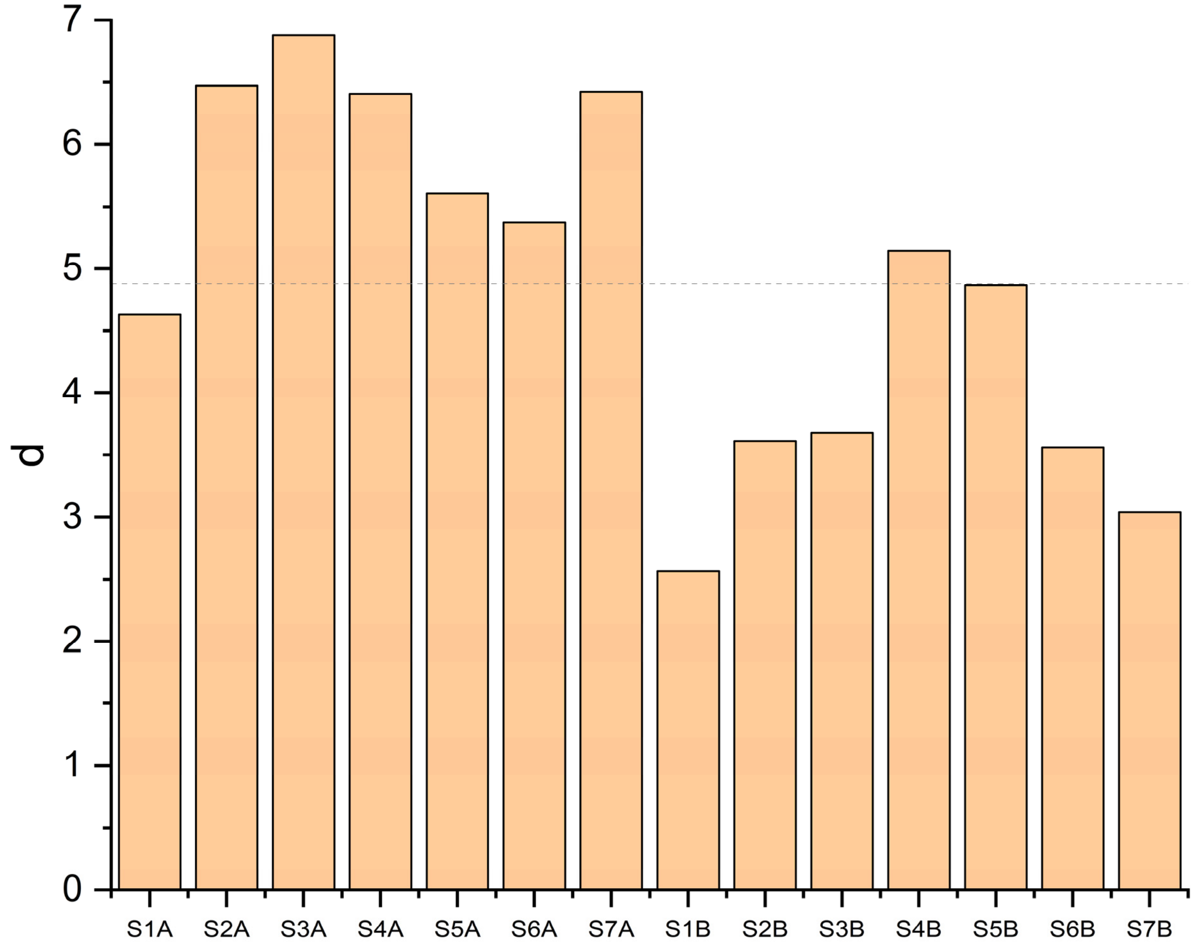

Figure S1. Values of d at each station. Note: A—stations in 2011; B—stations in 2012; broken line—mean value.

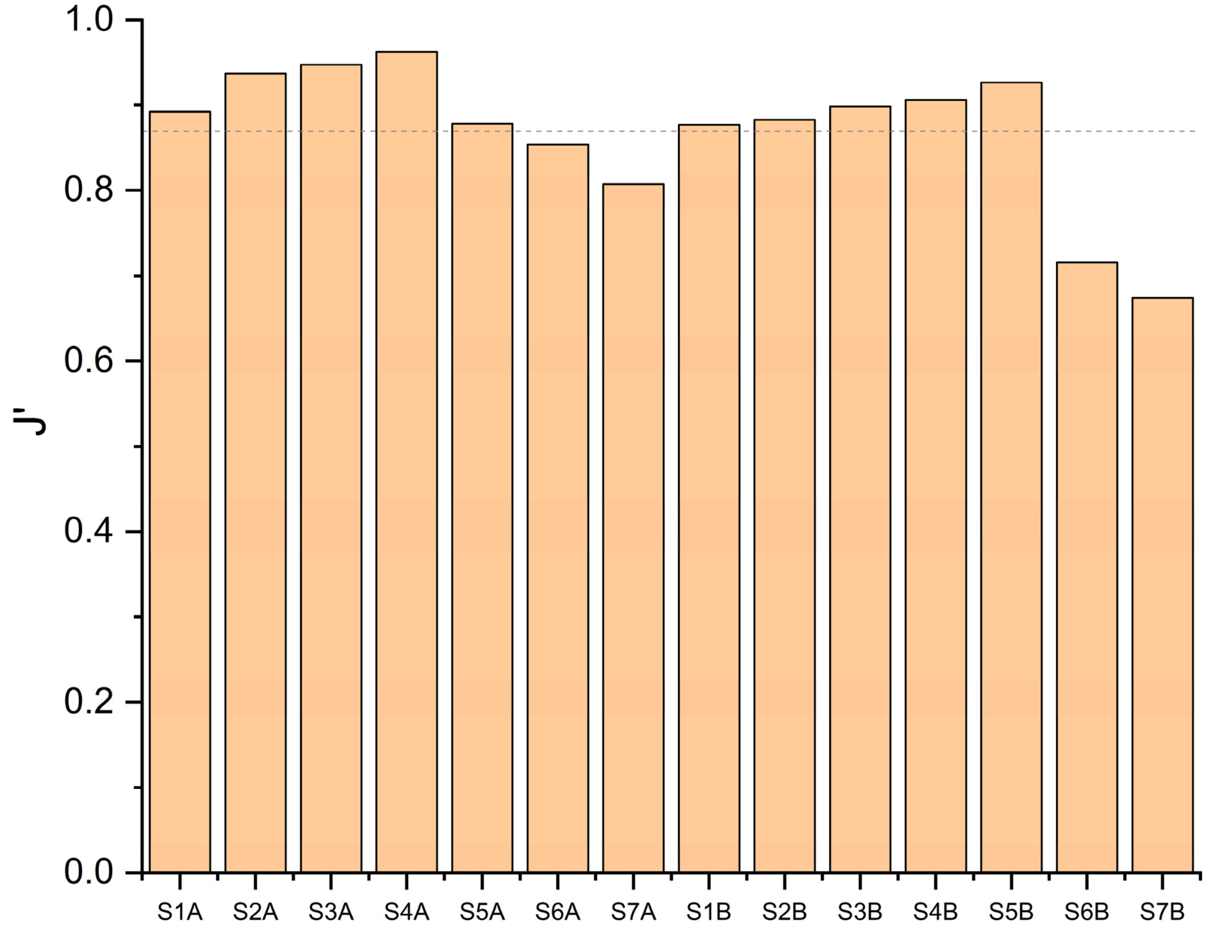

Figure S2. Values of J’ at each station. Note: A—stations in 2011; B—stations in 2012; broken line—mean value.

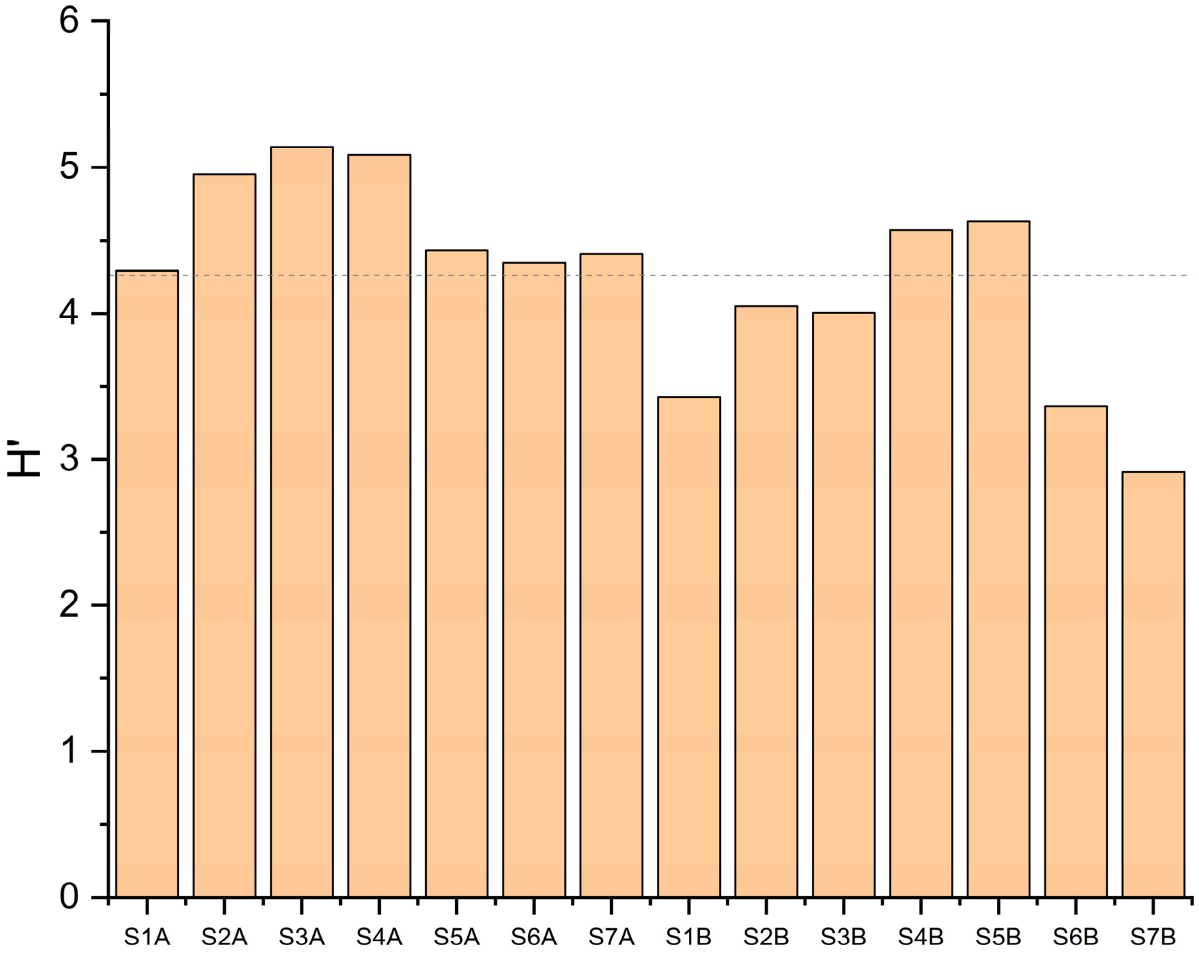

Figure S3. Values of H′ at each station. Note: A—stations in 2011; B—stations in 2012; broken line—mean value.

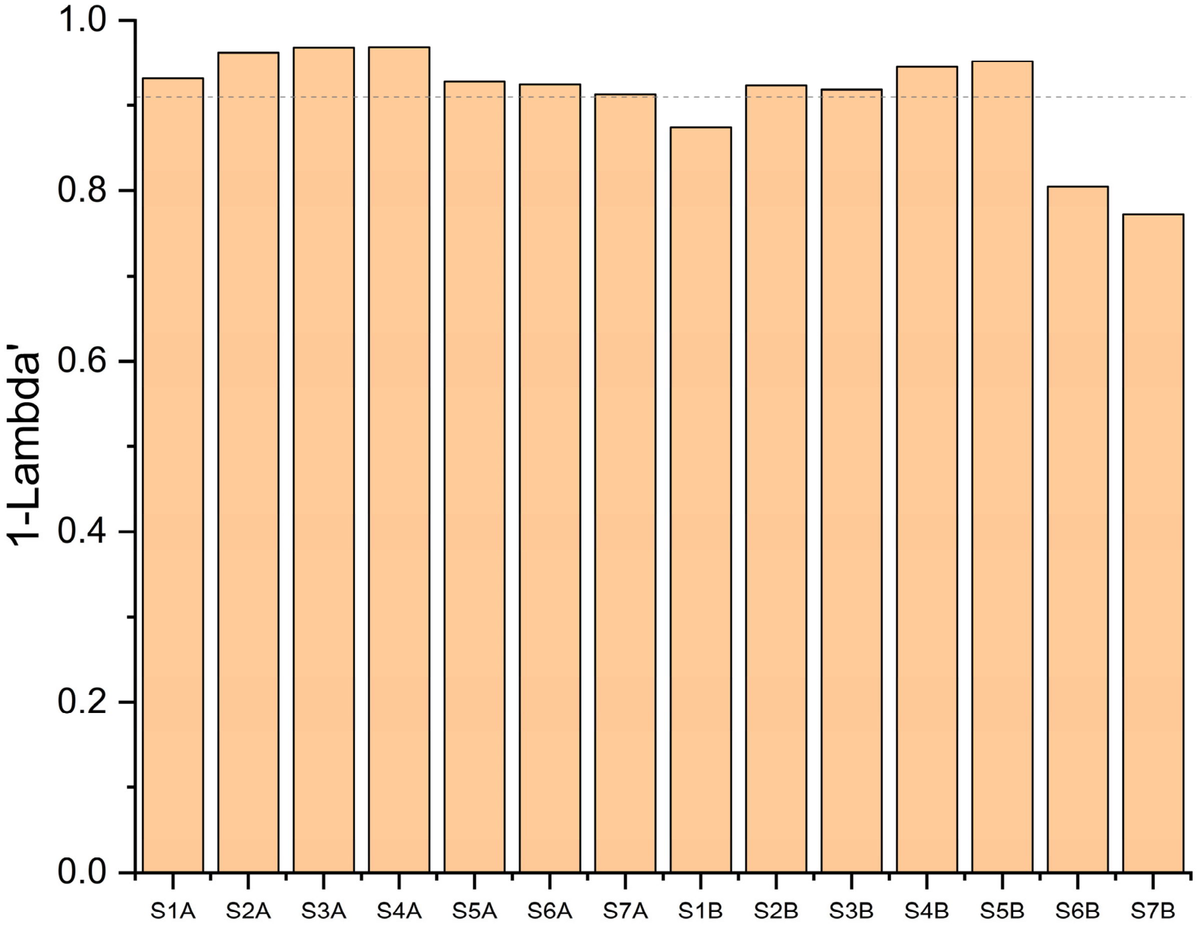

Figure S4. Values of 1-Lambda' at each station. Note: A—stations in 2011; B—stations in 2012; broken line—mean value.

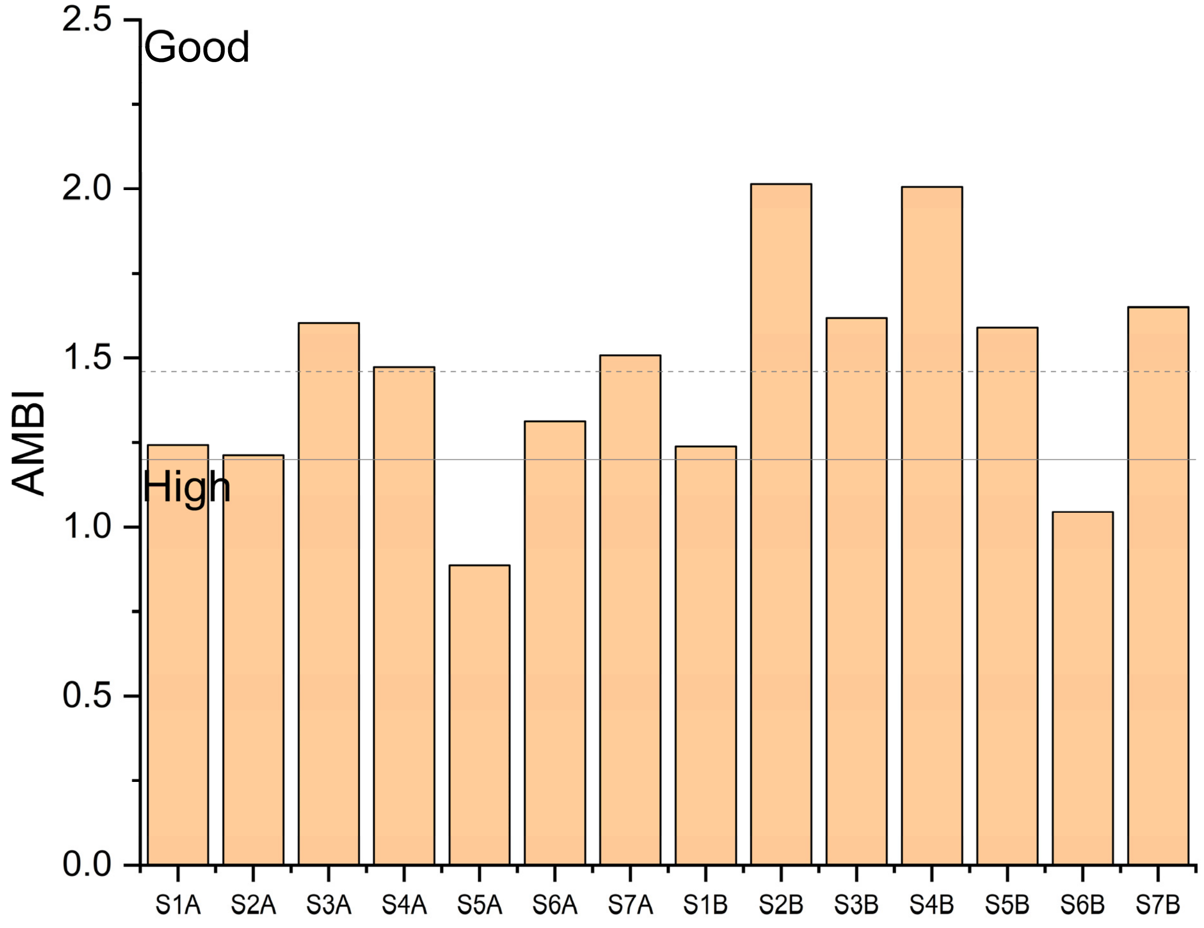

Figure S5. Values of AMBI and benthic ecological quality at each station. Note: A—stations in 2011; B—stations in 2012; broken line—mean value.

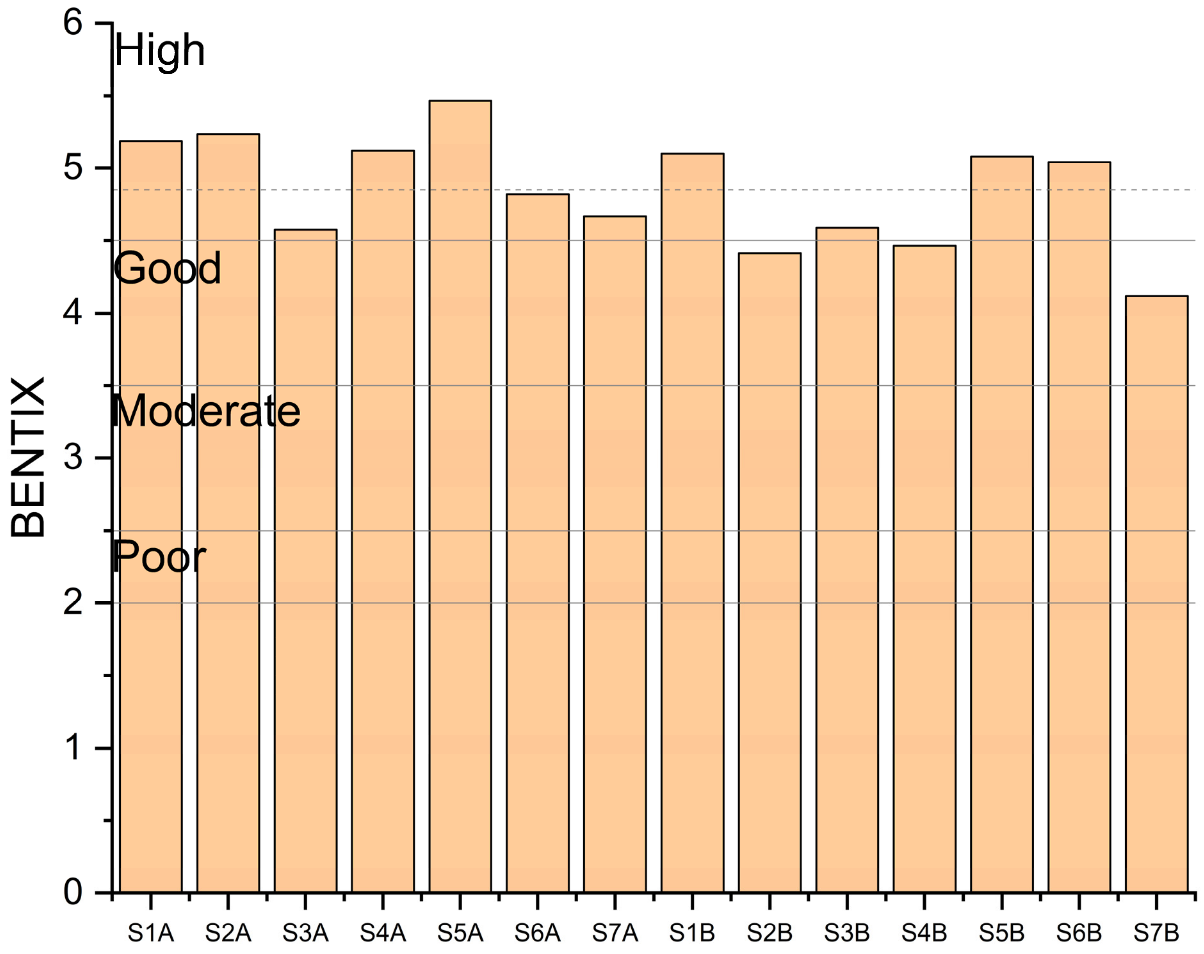

Figure S6. Values of BENTIX and benthic ecological quality at each station. Note: A—stations in 2011; B—stations in 2012; broken line—mean value.

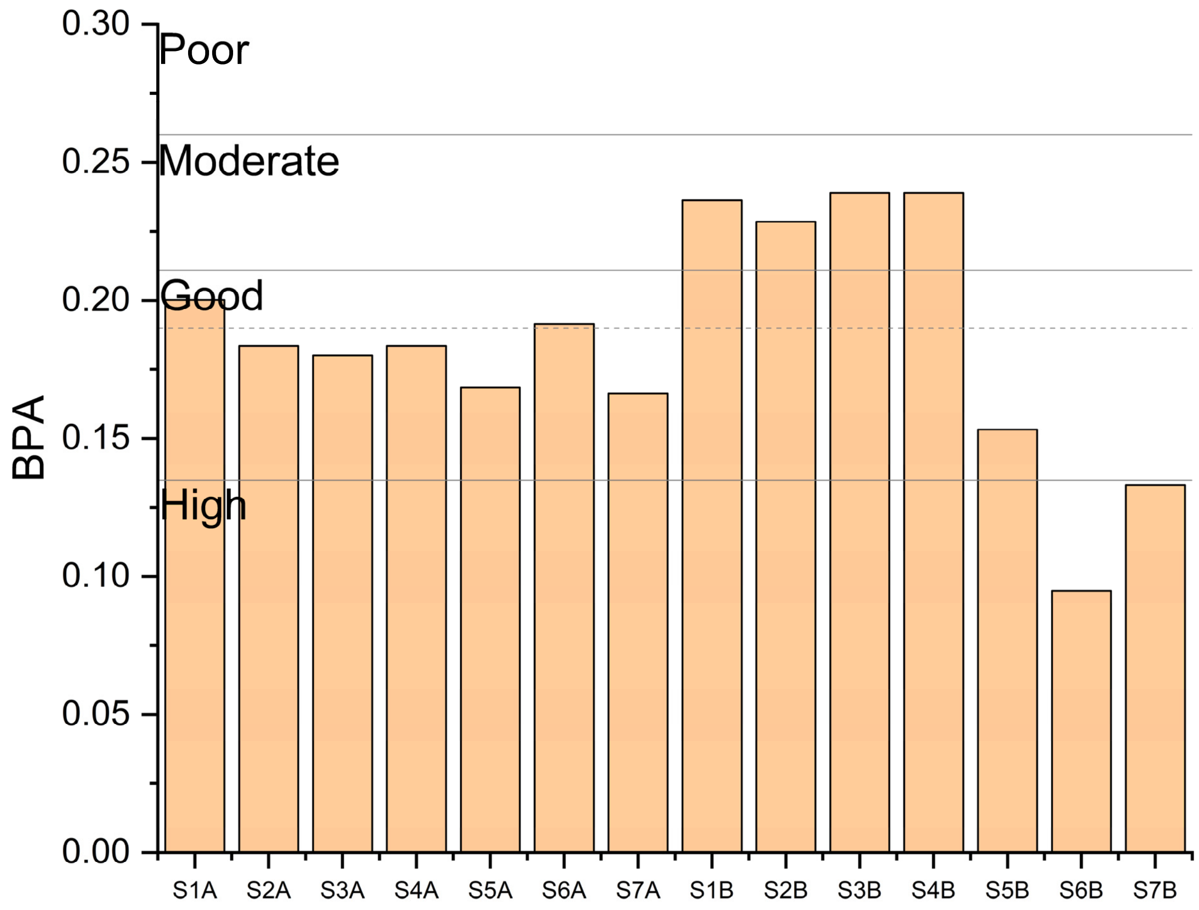

Figure S7. Values of BPA and benthic ecological quality at each station. Note: A—stations in 2011; B—stations in 2012; broken line—mean value.

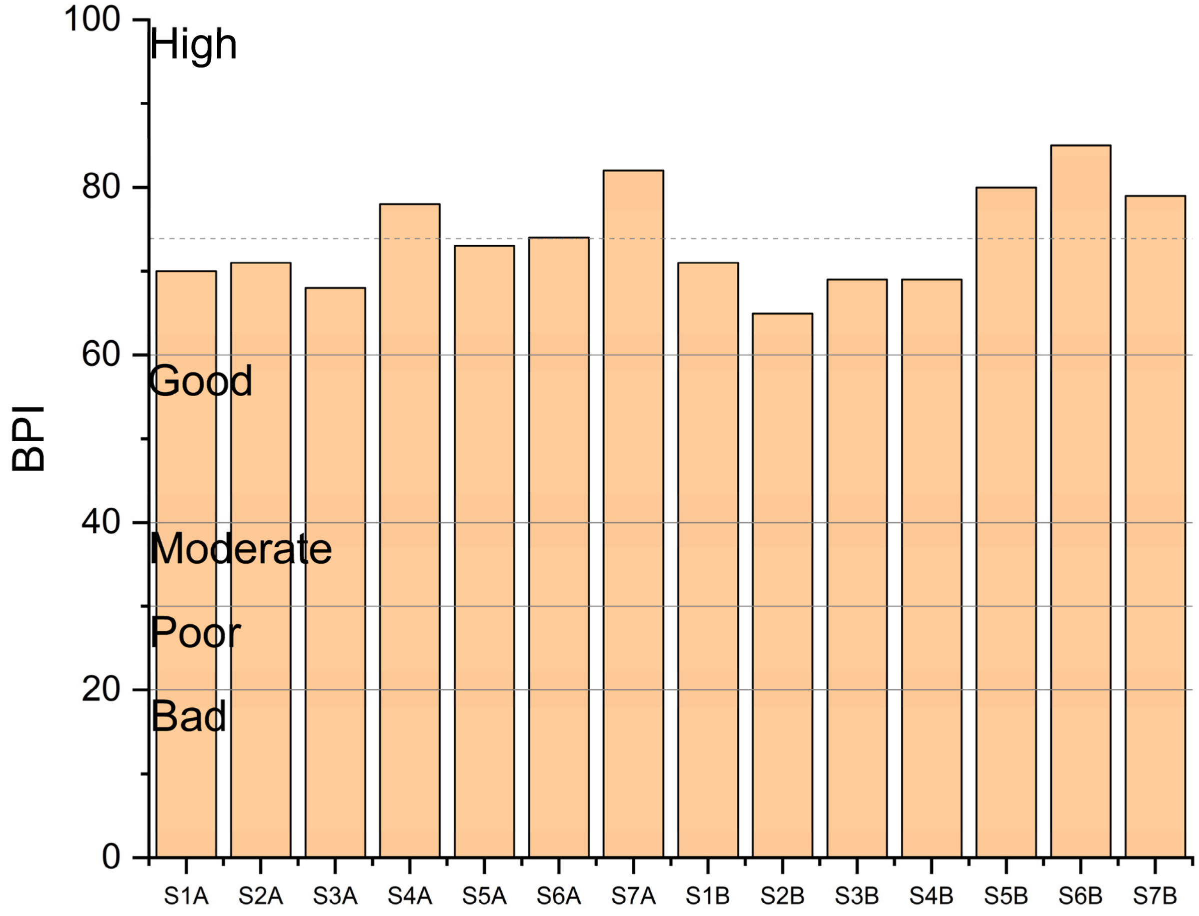

Figure S8. Values of BPI and benthic ecological quality at each station.

Note: A—stations in 2011; B—stations in 2012; broken line—mean value.

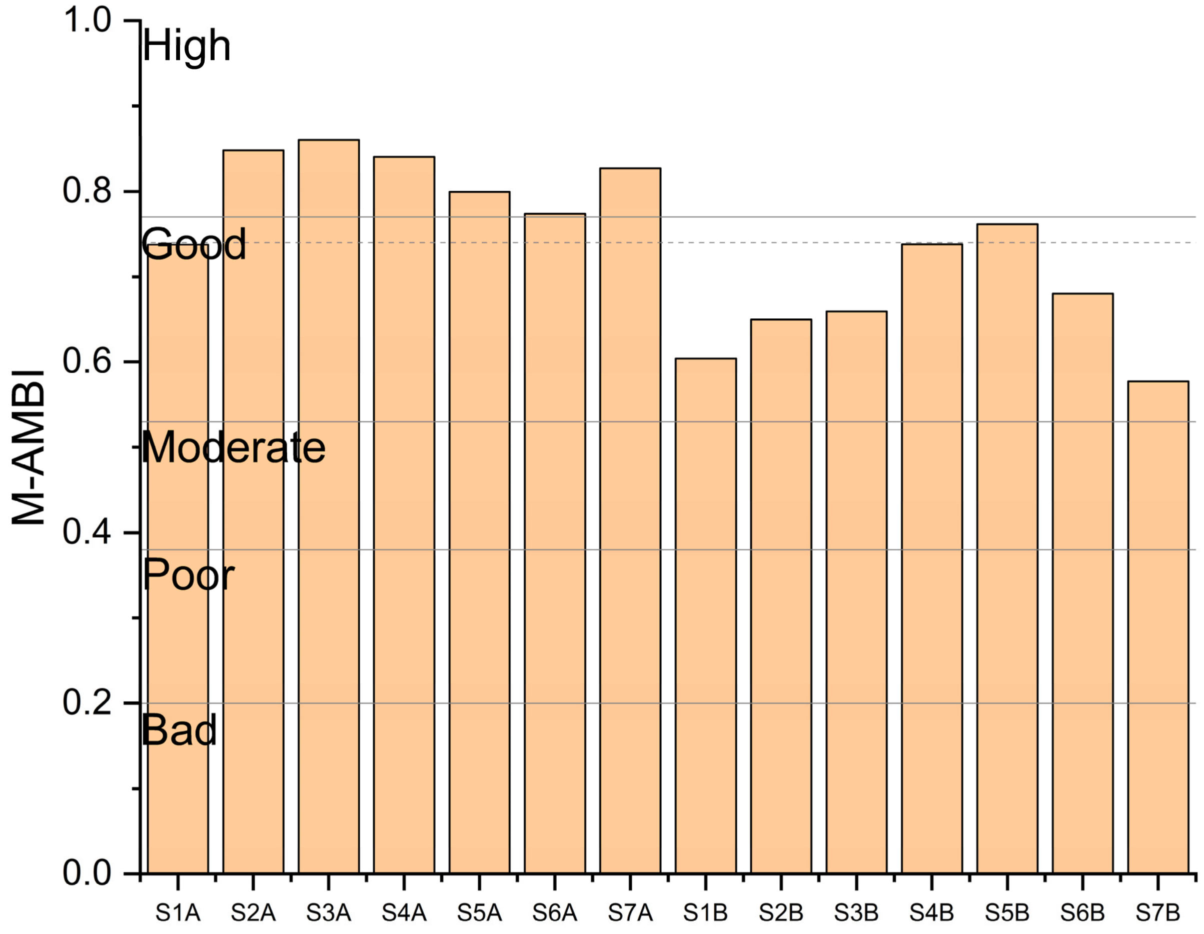

Figure S9. Values of M-AMBI and benthic ecological quality at each station. Note: A—stations in 2011; B—stations in 2012; broken line—mean value.
